# Gene expression analysis in *Fmr1*KO mice identifies an immunological signature in brain tissue and mGluR5-related signaling in primary neuronal cultures

**DOI:** 10.1186/s13229-015-0061-9

**Published:** 2015-12-21

**Authors:** Daria Prilutsky, Alvin T. Kho, Nathan P. Palmer, Asha L. Bhakar, Niklas Smedemark-Margulies, Sek Won Kong, David M. Margulies, Mark F. Bear, Isaac S. Kohane

**Affiliations:** Department of Biomedical Informatics, Harvard Medical School, Boston, MA USA; Children’s Hospital Informatics Program, Boston Children’s Hospital, Boston, MA USA; Picower Institute for Learning and Memory, Massachusetts Institute of Technology, Cambridge, MA USA; Department of Immunology, Harvard Medical School, Boston, MA USA; Department of Pediatrics, Harvard Medical School, Boston, MA USA; Divisions of Genetics and Developmental Medicine, Department of Pediatrics, Harvard Medical School, Boston, MA USA

**Keywords:** Fragile X syndrome, Murine model, Gene expression, Neuronal cultures, Brain

## Abstract

**Background:**

Fragile X syndrome (FXS) is a neurodevelopmental disorder whose biochemical manifestations involve dysregulation of mGluR5-dependent pathways, which are widely modeled using cultured neurons. In vitro phenotypes in cultured neurons using standard morphological, functional, and chemical approaches have demonstrated considerable variability. Here, we study transcriptomes obtained in situ in the intact brain tissues of a murine model of FXS to see how they reflect the in vitro state.

**Methods:**

We used genome-wide mRNA expression profiling as a robust characterization tool for studying differentially expressed pathways in fragile X mental retardation 1 (*Fmr1*) knockout (KO) and wild-type (WT) murine primary neuronal cultures and in embryonic hippocampal and cortical murine tissue. To study the developmental trajectory and to relate mouse model data to human data, we used an expression map of human development to plot murine differentially expressed genes in KO/WT cultures and brain.

**Results:**

We found that transcriptomes from cell cultures showed a stronger signature of *Fmr1*KO than whole tissue transcriptomes. We observed an over-representation of immunological signaling pathways in embryonic *Fmr1*KO cortical and hippocampal tissues and over-represented mGluR5-downstream signaling pathways in *Fmr1*KO cortical and hippocampal primary cultures. Genes whose expression was up-regulated in *Fmr1*KO murine cultures tended to peak early in human development, whereas differentially expressed genes in embryonic cortical and hippocampal tissues clustered with genes expressed later in human development.

**Conclusions:**

The transcriptional profile in brain tissues primarily centered on immunological mechanisms, whereas the profiles from cell cultures showed defects in neuronal activity. We speculate that the isolation and culturing of neurons caused a shift in neurological transcriptome towards a “juvenile” or “de-differentiated” state. Moreover, cultured neurons lack the close coupling with glia that might be responsible for the immunological phenotype in the intact brain. Our results suggest that cultured cells may recapitulate an early phase of the disease, which is also less obscured with a consequent “immunological” phenotype and in vivo compensatory mechanisms observed in the embryonic brain. Together, these results suggest that the transcriptome of cultured primary neuronal cells, in comparison to whole brain tissue, more robustly demonstrated the difference between *Fmr1*KO and WT mice and might reveal a molecular phenotype, which is typically hidden by compensatory mechanisms present in vivo. Moreover, cultures might be useful for investigating the perturbed pathways in early human brain development and genes previously implicated in autism.

**Electronic supplementary material:**

The online version of this article (doi:10.1186/s13229-015-0061-9) contains supplementary material, which is available to authorized users.

## Background

Fragile X syndrome (FXS) is the most common inherited form of mental retardation and the most common genetic cause of autism. It is caused by loss-of-function mutations in the gene fragile X mental retardation 1 (*Fmr1*) and a consequent loss of its product, fragile X mental retardation protein (FMRP) [1–3]. The pathophysiology of FXS involves dysregulation of numerous pathways. Recent studies have begun to provide insights into the biology of FMRP, and the results converged on metabotropic glutamate receptor (mGluR) signaling theory [1, 2]. Huber et al. discovered that a form of synaptic plasticity, mGluR-dependent long-term synaptic depression (mGluR-LTD) was exaggerated in *Fmr1* knockout mice (*Fmr1*KO) [4]. This discovery led to the mGluR theory of FXS [1], which suggests that many of its clinical features are due to exaggerated responses to activation of mGluR5. This theory was validated when multiple FXS phenotypes were rescued in *Fmr1*KO mice by reducing the production of mGluR5 protein [5–7].

Neurons from *Fmr1*KO mice and from patients with FXS consistently have increased spine densities, as well as longer spines, reminiscent of immature filopodia [5, 8–11]. Additional synaptic phenotypes, including hyper-connectivity and exaggerated responses to mGluR5 activation, are also found in *Fmr1*KO mice [4, 5]. These phenotypes can be recapitulated in cultured neurons [12, 13]. They can be corrected by treatment with mGluR5 antagonists in both cultured neurons and mice [5, 13]. Thus, in vitro models derived from *Fmr1*KO mice are reasonable platforms for modeling synaptic alterations occurring in FXS. Whether these in vitro models also mirror in vivo whole-brain transcriptional regulation has been less studied.

Animal models may be useful for studying the mechanism of disease in FXS, but current methods for characterizing neuronal phenotypes in these models may not be sufficiently robust for high-throughput pre-clinical screening of potential drugs. Furthermore, the molecular phenotypes are themselves heterogeneous. Therefore, identifying stable molecular phenotypes and finding a suitably robust characterization assay is a high priority.

Recent work has shown that RNA expression signatures can identify shared subsets of pathogenic pathways and produce short lists of affected marker genes in a variety of diseased tissues [14, 15]. Done correctly, this process can define a transcriptomic landscape of diseases and tissues and provide directionality with which to measure perturbations “to” and “from” the diseased status. Shared or partially overlapping mechanisms underlie complex phenotypes and may constitute a smaller set of pathways than the number of genetic variants or the genes that contain them. We hypothesized that genome-wide transcriptomic arrays would provide more accurate phenotypic readouts across various tissues and that subsequent comprehensive pathway-level analysis could pinpoint mechanisms relevant to a disease, whereas other morphological methods provide limited phenotypic information.

Our original motivation was to determine how well cell-culture transcriptomes recapitulate differences found in situ in the intact brain tissues of a murine model of FXS. Here, we studied in vitro cultured hippocampal and cortical neurons from *Fmr1*KO and wild-type (WT) mice and compared them to primary brain tissue. We also compared the location of each of these transcriptomes in the human transcriptomic developmental trajectories to determine how the *Fmr1*KO state affected the transcriptomic “age” [16, 17] in situ and in culture. We found that this examination of transcriptional changes is able to provide greater resolution on the differences between disease and wild-type conditions.

## Methods

### Ethics statement

All animal experiments were conducted in accordance with the rules and regulations of the Institutional Animal Care and Use Committee at the Massachusetts Institute of Technology (MIT). *Fmr1*KO mice were originally obtained from The Jackson Laboratory (Bar Harbor, ME). The strain was maintained on a C57BL/6 background for at least six generations at MIT. All experiments were performed blind to genotype and were carried out on ex vivo brain tissue following euthanasia. Euthanasia methods are compatible with the recommendations of the Panel on Euthanasia of the American Veterinary Association.

### Neuronal culture, tissue isolations, and RNA extraction

Hippocampal and cortical neuron cultures were prepared from C57BL/6 congenic WT and *Fmr1*KO embryos at 17–18 days post-conception (E17–E18) in parallel and allowed to mature for 14 days in vitro (DIV) as previously described [18]. Five pairs of biological replicates for WT and *Fmr1*KO were processed on the same day. Cells were seeded at a concentration of 150,000/mL and maintained in Neurobasal media supplemented with B27, penicillin/streptomycin, and glutamax (Invitrogen) according to the manufacturer’s instructions. Cells were harvested on day 14 in PBS and snap frozen using dry ice. The estimated percentage of glial cells at 14 DIV is ~30 %. In parallel to the establishment of primary cultures, ~10 % of hippocampal and cortical tissues were dedicated to tissue analyses and stored in RNA*later* for stabilization until further processing.

Four separate sample types were used per genotype (WT or KO): primary cortex (*n* = 5), primary hippocampus (*n* = 6), cortical culture (*n* = 5), and hippocampal culture (*n* = 5). Use of duplicates resulted in 42 samples. RNA was extracted using the miRNeasy Mini Kit (Qiagen). Eluted RNA was analyzed on a NanoDrop ND-1000 Spectrophotometer and an Agilent 2100 Bioanalyzer (OD260/280 ratio: 1.8–2.2; RNA Integrity Number >8).

### Transcriptome profiling using microarrays

A total of 100 ng of RNA was processed using Affymetrix (Affymetrix Inc, Santa Clara, CA) protocols and kits, which were used to generate biotinylated amplified RNA (aRNA) and for hybridization, staining, and scanning of arrays (GeneChip 3’IVT Express Kit and GeneChip Expression Wash, Stain and Scan protocol). Total RNA was reverse-transcribed to synthesize double-stranded cDNA using T7 oligo(dT) primers and then transcribed in vitro into biotin-modified aRNA with IVT Labeling Master Mix. The aRNA was purified, quantified, and fragmented. Fragmented aRNA was hybridized onto Affymetrix Mouse Genome 430 2.0 arrays and scanned on an Affymetrix GeneChip scanner 3000 at 2.5 μm resolution. Microarray data are deposited at the Gene Expression Omnibus database (GSE71034).

### Microarray analysis and pathway-level analysis

Expression values were extracted and normalized from .CEL files using the Affy package and the robust multi-array average (RMA) method in R/BioConductor (http://www.bioconductor.org), returning the measured gene expression signal of each microarray gene probe in a logarithmic base 2 scale. Differential gene expression analysis was performed using a linear regression model (lmFit) as implemented in the limma package in R/BioConductor, and significant differentially expressed probes (*p* < 0.05) were extracted. We used the Database for Annotation, Visualization and Integrated Discovery (DAVID, http://david.abcc.ncifcrf.gov) to identify enriched pathways in differentially expressed genes at a Fisher exact *p* value threshold (EASE score) less than 0.1, which were annotated with Entrez IDs. We annotated 45,101 probes with 21,141 unique Entrez Gene IDs by selecting the smallest Entrez Gene ID for any probe originally assigned to >1 ID. For pathway-level analysis, we focused on the Kyoto Encyclopedia of Genes and Genomes (KEGG) pathways. Additionally, we performed pathway-level analysis using MetaCore software (Gene Go Inc., St. Joseph, MI). Enrichment analysis consisted of mapping Entrez Gene IDs of differentially expressed genes in culture and in brain onto IDs in entities of built-in functional ontologies represented in MetaCore by process networks and diseases to identify biological processes that were over-represented.

Principal component analysis (PCA) was used to characterize the directions of maximal transcriptomic variance in the whole dataset [19–21]. PCA was performed on an RMA-normalized, ranked, and standardized matrix (mean zero and variance one) of 42 samples (45,101 probes represented on the array). The percentage variance captured by each of the first two principal components (PCs) were 61.3 % by PC1 and 11 % by PC2.

We examined the over-representation of differentially expressed genes in *Fmr1*KO hippocampal and cortical culture and embryonic brain tissues and three lists of genes associated with defects in cognitive development (Additional file 1: Table S1):

1. MGI genes associated with the behavioral/neurological phenotype in mice [22]
  A search for the term “MP:0005386” in the JAX lab mouse database (http://www.informatics.jax.org/marker/) returned 3005 unique gene symbols, which are 2622 unique genes mapped to Entrez Gene IDs, out of which 2462 were measured on the Affymetrix Mouse Genome 430 2.0 microarray.
2. Human genes from SFARI [23]: 705 genes, out of which 645 were mapped to mouse genes
3. FMRP binding targets as identified by Ascano et al. [24]: 939 genes, out of which 898 were mapped to mouse genes

### Human brain transcriptome data processing and integration with mouse data

A spatio-temporal transcriptome of the developing human brain (human brain transcriptome (HBT)) has been described previously [25], and these data are publicly available in NIH’s Gene Expression Omnibus (http://www.ncbi.nlm.nih.gov/geo) as GSE25219. These samples were profiled on Affymetrix Human Exon 1.0 ST Array, and we used the transcript (gene)-to-sample series matrix for the further analysis. In order to integrate mouse and human gene expression data, we converted 45,101 probes from the Mouse Affymetrix 430 Plus 2.0 array to 21,141 unique Entrez Gene IDs by selecting the smallest Entrez Gene ID for any probe originally assigned to >1 ID. For each differential comparison in mouse data, probe-to-gene unique-ing was done by picking the probe with smallest *p* value to represent the gene. After downloading human brain transcriptome data from GEO, we converted 17,565 probes to 16,492 unique min human Entrez Gene IDs, 14,653 unique corresponding min homologous mouse Entrez Gene IDs, and 13,830 unique mouse Entrez Gene IDs in common with the Affymetrix Mouse Genome 430 2.0 microarray. We restricted analysis of human data to hippocampus (HIP) and neocortex (NCX) at 15 developmental stages. NCX data included 11 areas collectively referred as the NCX region. In the NCX data for each probe, we computed the sum of coefficients of variance (coefvar) in stages 2–15. For each gene, we selected the probe with the minimal sum of coefvar for probe-to-gene unique-ing. We used the same probes for HIP probe-to-gene unique-ing. In all analyses comparing the two datasets above, we restricted data to 13,830 mouse genes in common between the datasets.

PCA of genes in the sample space was performed on 936 human NCX samples and 82 human HIP samples separately, and k-means clustering (*k* = 3) was used to generate temporal clusters. Before the PCA of human data, each individual sample, a vector of 13,830 genes, was first normalized to mean zero and variance one. Principal components were invariant under affine transformations.

## Results

### Gene expression profiles in embryonic brain tissue capture fewer genotype-based differences than primary neuronal cultures

In order to find gene expression differences between the FXS and healthy states, we extracted embryonic hippocampal and cortical tissues from *Fmr1*KO and WT mice at the age of 17–18 days post-conception (E17–E18). One portion of the tissue was used to create primary neuronal cultures, and the other was used directly in the study of brain tissue.

**Fig. 1:**
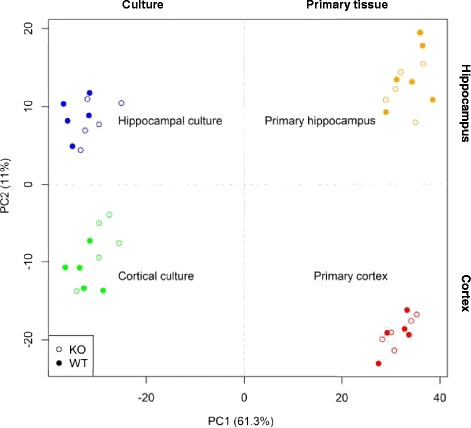
Principal component analysis (PCA) of global transcriptional profiles in murine *Fmr1*KO/WT culture and brain tissues. PCA captures the differences between primary tissue or culture type and region of the brain as identified by expression levels of 45,101 probes in 42 samples. The scatter indicates that gene expression profiles of cortical and hippocampal neuronal cultures captured the difference between genotypes

Total RNA (100 ng) from each sample was extracted, amplified, labeled, and hybridized on Mouse Genome 430 2.0 Arrays (Affymetrix, Santa Clara, CA). Principal component analysis (PCA) was used to characterize the directions of maximal transcriptomic variance in the whole dataset (Fig. 1, each dot signifies a sample). The PCA captured 72.4 % of total variation in the data with the first two principal components (PCs) – 61.3 % captured by PC1 and 11.1 % by PC2. This analysis indicated that the greatest difference was observed between cultured cells and whole tissue, representing the effects of preparation, growth, and differentiation. The second greatest difference was between hippocampal and cortical tissue, representing different brain regions. In addition, Fig. 1 shows that transcriptomic scale differences between two genotypes *Fmr1*KO and WT are greater in the cultured neurons versus whole embryonic cortical and hippocampal tissue. Additional file 2: Table S2 shows the metrics used to represent the difference between *Fmr1*KO and WT mice. The PC1-axis centroid distance between KO and WT clusters in both cultures was at least three times greater than the distance between KO and WT in primary brain. In addition, the number of significant differentially expressed genes at an unadjusted *p* value of 0.05 was almost four times greater in vitro than in whole tissues. Our analysis identified differentially expressed genes (*p* value <0.05) in *Fmr1*KO versus WT as follows: 2648 in hippocampal culture, 3372 in cortical culture, 726 in primary hippocampus, and 866 in primary cortex (Additional file 3: Table S3). *Fmr1* was the most significantly down-regulated gene in all systems studied (~twofold down-regulation with *p* value <3.75E−15).

### Enriched biological pathways converge on mGluR5-downstream signaling pathways in cultured neurons, while the pathways in embryonic brain tissues center on genes associated with immunological signaling

Differential gene expression analysis was performed using a linear regression model as implemented in the limma library package in R/BioConductor, and significant differentially expressed probes (*p* < 0.05) were extracted. We used unadjusted *p* value < 0.05 for significance threshold. Next, Kyoto Encyclopedia of Genes and Genomes (KEGG) pathway-enrichment analysis of differentially expressed genes between KO and WT was performed using the Database for Annotation, Visualization and Integrated Discovery (DAVID; Table 1 and Additional file 4: Table S4).

**Table 1:** Enriched pathways in differentially expressed genes in brain and primary cultures of *Fmr1*KO mice

KEGG pathway (ENTREZ ID as input)	*p* value	No. of genes	Fold enrichment
Hippocampal culture			
*mmu04910:Insulin signaling pathway*	*0.001*	*35*	*1.748*
*mmu03040:Spliceosome*	*0.001*	*32*	*1.801*
*mmu00900:Terpenoid backbone biosynthesis*	*0.002*	*8*	*3.796*
*mmu04720:Long-term potentiation*	*0.003*	*20*	*2.013*
*mmu04120:Ubiquitin mediated proteolysis*	*0.008*	*32*	*1.586*
*mmu04360:Axon guidance*	*0.009*	*31*	*1.584*
*mmu03010:Ribosome*	*0.011*	*22*	*1.740*
*mmu05211:Renal cell carcinoma*	*0.013*	*19*	*1.803*
*mmu04540:Gap junction*	*0.017*	*21*	*1.701*
*mmu04722:Neurotrophin signaling pathway*	*0.017*	*29*	*1.541*
*mmu00230:Purine metabolism*	*0.022*	*33*	*1.461*
*mmu00650:Butanoate metabolism*	*0.029*	*11*	*2.088*
*mmu04012:ErbB signaling pathway*	*0.031*	*21*	*1.603*
*mmu05215:Prostate cancer*	*0.038*	*21*	*1.567*
*mmu03018:RNA degradation*	*0.039*	*15*	*1.748*
*mmu03020:RNA polymerase*	*0.040*	*9*	*2.214*
mmu00790:Folate biosynthesis	0.070	5	3.019
mmu04914:Progesterone-mediated oocyte maturation	0.078	19	1.485
mmu04150:mTOR signaling pathway	0.080	13	1.661
mmu00240:Pyrimidine metabolism	0.088	20	1.444
mmu05220:Chronic myeloid leukemia	0.088	17	1.506
Cortical culture			
*mmu04622:RIG-I-like receptor signaling pathway*	*0.001*	*23*	*2.027*
*mmu03040:Spliceosome*	*0.001*	*37*	*1.686*
*mmu04722:Neurotrophin signaling pathway*	*0.006*	*36*	*1.548*
*mmu04144:Endocytosis*	*0.012*	*48*	*1.395*
*mmu04660:T cell receptor signaling pathway*	*0.013*	*31*	*1.529*
*mmu04010:MAPK signaling pathway*	*0.016*	*63*	*1.308*
*mmu04120:Ubiquitin mediated proteolysis*	*0.018*	*36*	*1.444*
*mmu05220:Chronic myeloid leukemia*	*0.029*	*22*	*1.577*
mmu04720:Long-term potentiation	0.052	19	1.548
mmu00534:Heparan sulfate biosynthesis	0.062	9	2.016
mmu03450:Non-homologous end-joining	0.076	6	2.481
mmu04062:Chemokine signaling pathway	0.080	41	1.267
mmu05210:Colorectal cancer	0.096	22	1.391
Hippocampus in situ			
*mmu04144:Endocytosis*	*0.013*	*14*	*2.133*
*mmu04060:Cytokine-cytokine receptor interaction*	*0.013*	*16*	*1.986*
*mmu05322:Systemic lupus erythematosus*	*0.014*	*8*	*3.088*
*mmu04514:Cell adhesion molecules (CAMs)*	*0.017*	*11*	*2.348*
*mmu05320:Autoimmune thyroid disease*	*0.028*	6	*3.451*
mmu05330:Allograft rejection	0.051	5	3.523
mmu04672:Intestinal immune network for IgA production	0.082	5	2.998
mmu04610:Complement and coagulation cascades	0.099	6	2.415
Cortex in situ			
*mmu00590:Arachidonic acid metabolism*	*0.016*	*9*	*2.727*
*mmu04210:Apoptosis*	*0.029*	*9*	*2.439*
mmu04640:Hematopoietic cell lineage	0.059	8	2.275
mmu04060:Cytokine-cytokine receptor interaction	0.064	16	1.623
mmu00601:Glycosphingolipid biosynthesis	0.083	4	3.838

Italics highlight pathways with *p* values <0.05

The KEGG pathways that were most significantly enriched with the differentially expressed genes in *Fmr1*KO cultured cells and isolated embryonic hippocampal and cortical tissues were related to synaptic signaling, immunological response, and cell-cell interactions. Sixteen pathways were significantly enriched in *Fmr1*KO hippocampal culture compared to WT equivalent cultures (Table 1). Six have been extensively implicated in synaptic signaling. They include “insulin signaling pathway” (*p* value = 0.001), “long-term potentiation (LTP)” (*p* value = 0.003), “axon guidance” (*p* value = 0.009), “gap junction” (*p* value = 0.017), “neurotrophin signaling pathway” (*p* value = 0.017) and “mTOR signaling pathway” (*p* value = 0.08). Eight pathways were significantly enriched in *Fmr1*KO cortical cultures (Table 1), including “neurotrophin signaling pathway” (*p* value = 0.006), “MAPK signaling pathway” (*p* value = 0.016), and “long-term potentiation” (*p* value = 0.052). Five pathways were significantly enriched in *Fmr1*KO in situ hippocampus studies relating to immunological response (such as “cytokine-cytokine receptor interaction” (*p* value = 0.013)) and cell adhesion (“cell adhesion molecules” (*p* value = 0.017)). Two pathways were significantly enriched in *Fmr*1KO primary cortex. One of them was “arachidonic acid metabolism” (*p* value = 0.016), which has a role in inflammation and formation of an important group of inflammatory mediators. Several synaptic mGluR5-coupled signaling pathways perturbed in KO versus WT in cortical and hippocampal neuronal cultures include genes that may have roles in FMRP-regulated mRNA translation at the synapse (Additional file 5: Table S5). Importantly, the signature in embryonic brain tissue was primarily immunological, compared to a primarily synaptic signature in the cultured samples.

To validate and reproduce our findings, we also performed pathway-enrichment analysis on differentially expressed genes in culture and in brain using a separate tool, MetaCore, with similar outcomes. Networks associated with neuronal processes were over-represented in cultures (Development_Neurogenesis_Axonal Guidance, Additional file 6: Figure S1A). In contrast, networks associated with immune disorders were over-represented in brain samples (Autoimmune Diseases, Additional file 6: Figure S1B).

In addition to analyzing each system separately, we also examined the overlap of pathways and commonalities between embryonic brain and cultures and between two different brain regions (cortex and hippocampus; Additional file 7: Table S7). The commonalities between cortical and hippocampal cultures converged on pathways such as “ribosome,” “neurotrophin signaling pathway,” “long-term potentiation,” “endocytosis,” and “spliceosome,” which mostly relate to synaptic signaling. In brain, we observed convergence on the “cell adhesion molecules” pathway between hippocampus and cortex.

We examined the over-representation of differentially expressed genes in *Fmr1*KO cultures and brain tissues for three gene sets associated with cognitive development defects: (1) Mouse Gene Informatics (MGI) genes associated with behavioral/neurological phenotypes [22]; (2) human autism candidate genes from Simons Foundation Autism Research Initiative (SFARI) [23]; and (3) FMRP binding targets [24] (Additional file 8: Table S8). The genes down-regulated in murine *Fmr1*KO cultures were enriched for MGI behavioral/neurological phenotype genes, human SFARI-related genes, and FMRP targets. However, the overlap between these three lists and differentially expressed genes in embryonic brain tissue was not significant (Additional file 9: Table S9, Additional file 10: Table S10 and Additional file 11: Table S11). We also found a significant overlap between SFARI and FMRP (*p* value = 4.41E−7), SFARI and MGI behavioral (*p* value < 2.2E−16), FMRP and MGI behavioral (*p* value = 1.03E−8) gene lists.

### Differentially expressed genes in *Fmr1*KO in cultured neurons are preferentially expressed in early human brain development, whereas genes in embryonic hippocampal and cortical tissues correspond more closely to later developmental stages

To identify the predominant gene clusters in mice that corresponded to developing human neocortex and hippocampus, we used PCA to compare 13,830 genes in sample space in the two datasets, respectively. Our reference model for development was the human neocortical and hippocampal transcriptome at 15 stages, profiled using Affymetrix Human Exon 1.0 ST Array as previously described [25]. We integrated this reference data with our mouse data, which comprised 21,141 unique genes. Of the 16,492 unique minimal human Entrez IDs present on Affymetrix Exon ST 1.0 array, 14,653 genes correspond with homologous mouse Entrez Gene ID and 13,830 unique genes are shared between the Human Exon 1.0ST and Mouse 430 2.0 arrays (Additional file 12: Table S12).

**Fig. 2:**
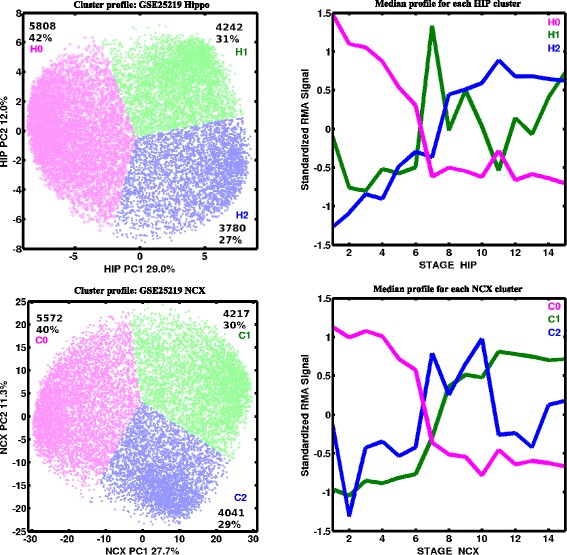
Integration of human GSE25219 and mouse data with cluster-representative profiles. The maps show gene-centric PCA (left panel) of 15 human developmental stages in hippocampus (*top*) and in cortex (*bottom*). Median profile for each cluster is represented on the right panel of the figure

Figure 2 shows the first two principal components for each gene (each dot signifies a gene). In the hippocampus, PC1 and PC2 captured 29 and 12 % of variation, respectively, and in the neocortex, they captured 27.7 and 11.3 % of variation, respectively. Visually, there appeared to be three large-scale patterns of co-expression in each of the developmental time series represented on PCA. Therefore, we used K-means clustering (*k* = 3) to clarify these clusters. The first cluster primarily had genes where the first PC was negative (PC1 < 0; H0 and C0 in the hippocampus and cortex respectively; magenta dots). Plotting these genes against developmental stage showed that they were typically up-regulated between the first and sixth stages of development and that their expression decreased subsequently (median value for each cluster based on RMA-normalized signal; right side of Fig. 2). Stages 1 to 7 are defined as fetal stages in Kang et al. [25]. Genes with a positive first PC1 (PC1 > 0) were subdivided to two clusters, cluster 1 (green dots, H1/C1) and cluster 2 (blue dots, H2/C2). Genes whose expression was higher during developmental stages 7–11 are represented by H1 and C2 (infancy and childhood). H2 and C1 correspond primarily to genes up-regulated during young, middle, and late adulthood. The border between H0/H1 and C0/C2 may be defined as birth, with its associated broad changes in gene expression. Overall, we observed that during human hippocampal development, 42 % (5808), 31 % (4242), and 27 % (3780) of total genes resided in H0, H1, and H2 clusters, respectively. During cortical development, 40 % (5572), 30 % (4217), and 29 % (4041) of total genes resided in the C0, C1, and C2 clusters, respectively.

**Fig. 3:**
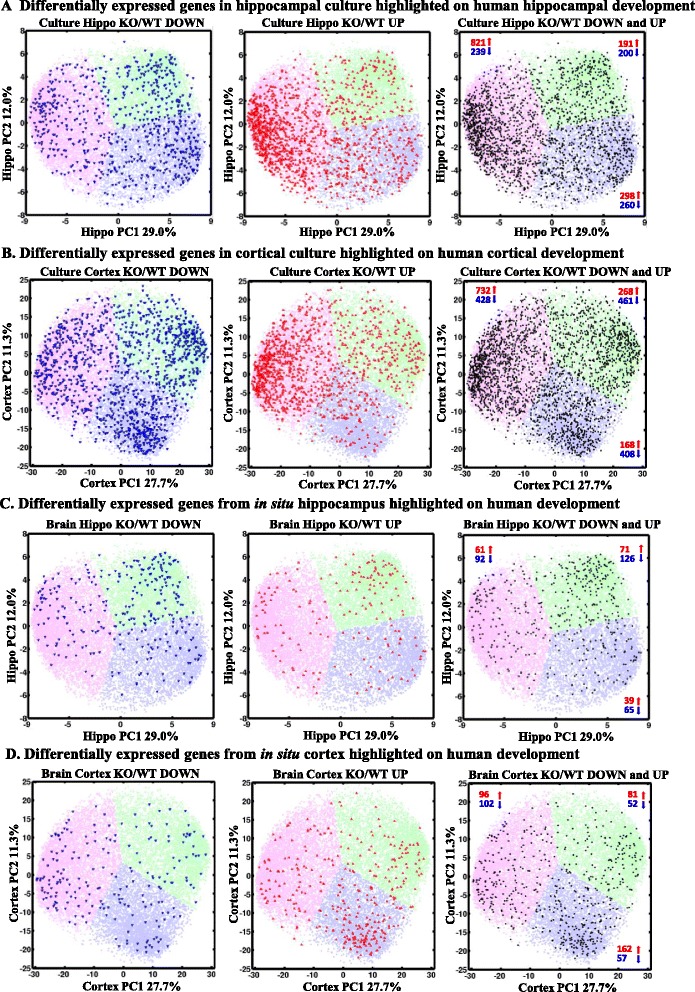
Differentially expressed murine genes highlighted on the human developmental transcriptome map. Differentially expressed genes in *Fmr1*KO **a** hippocampal and **b** cortical culture are expressed early in human development and expressed late in **c** in situ hippocampus and **d** in situ cortex (blue: down-regulated, red: up-regulated, black: combined)

To investigate the expression profiles of murine homologues in the human data, we highlighted differentially expressed murine genes onto the human developmental transcriptome map. Figure 3 shows differentially expressed genes (blue: down-regulated, red: up-regulated, black: combined) for hippocampal and cortical KO/WT cultures and for embryonic hippocampus and cortex tissue.

The majority of genes that were up-regulated in murine *Fmr1*KO cortical and hippocampal cultures segregated into cluster 0. This finding is indicated by the increased density of red dots (genes) at the 9 o’clock periphery of the upper panels in Fig. 3 and the highest percentage in the corresponding cluster in Table 2. These findings indicate that genes up-regulated in KO mice tended to have increased expression in early development (than later) of the corresponding brain area.

**Table 2:** Integration of human neocortex/hippocampus developmental genes and mouse differentially expressed genes in culture and brain

Case/cluster	H0	H1	H2	Case/cluster	C0	C1	C2
All genes	5808	4242	3780	All genes	5572	4217	4041
%	42	31	27	%	40	30	29
Differentially expressed genes in cultured cells							
Hippo culture KO/WT down	239	200	260	Cortex culture KO/WT down	428	461	408
%	34	29	37	%	33	36	31
Hippo culture KO/WT up	821	191	298	Cortex culture KO/WT up	732	268	168
%	63	15	23	%	63	23	14
Hippo culture KO/WT combined	1060	391	558	Cortex culture KO/WT combined	1160	729	576
%	53	19	28	%	47	30	23
Differentially expressed genes in primary brain tissue							
Hippo brain KO/WT down	92	126	65	Cortex brain KO/WT down	102	52	57
%	32.5	44.5	23	%	48.3	24.6	27.1
Hippo brain KO/WT up	61	71	39	Cortex brain KO/WT up	96	81	162
%	35.7	41.5	22.8	%	28.3	24	47.7
Hippo brain KO/WT combined	153	197	104	Cortex brain KO/WT combined	198	133	219
%	33.7	43.4	22.9	%	36	24.2	39.8

Next, we highlighted differentially expressed genes in embryonic brain tissue in human development (Fig. 3c, d). We observed that the majority of differentially regulated genes in murine *Fmr1*KO primary hippocampus segregated to Cluster H1, which represents a later stage in human development (Table 2). The majority of up-regulated genes in murine *Fmr1*KO primary cortex segregated to cluster 2 (C2). This finding indicates that the majority of differentially expressed genes (mostly up-regulated genes which partition to H1 and C2 in the hippocampus and cortex) in primary brain tissue tended to be expressed at higher levels in later development. Overall, differentially expressed genes in murine culture were over-represented for early expression in humans (cortical culture: 95 % CI = 1.28–1.53, OR = 1.4; hippocampal culture: CI = 1.5–1.83, OR = 1.66), while the differentially expressed genes in tissue were under-represented at early stages (cortex: 95 % CI = 0.69–0.99, OR = 0.83; hippocampus: 95 % CI = 0.57–0.85, OR = 0.69) and over-represented in later stages (Additional file 13: Table S13).

Next, we determined if enrichment of up-regulated genes in KO mouse CNS in the early phases of development was a consequence of common non-cell-type-specific cell-cycle changes. We used the DAVID program to perform a KEGG pathway-level analysis of the 732 C0- and 821 H0-related up-regulated genes (red clusters in Fig. 3a, b). This analysis showed that in addition to cell-cycle-related processes, there was an over-representation of processes such as the neurotrophin signaling pathway and the T cell receptor signaling pathway in the C0-related subset of genes. In addition, the insulin signaling pathway, gap junction, endocytosis, ErbB signaling pathways and axon guidance were over-represented in the H0-related subset of genes (Additional file 14: Table S14).

## Discussion

The *Fmr1*KO mouse is a valuable model for studying FXS and a subset of patients with autism. We selected to work with FXS mouse model as it constitutes one of the more established and studied models of the more homogeneous monogenic forms of autism. Even so, we accept that it might not be fully representative for autism, which exhibits striking heterogeneity.

We investigated whether transcriptomic changes in *Fmr1*KO embryonic brain tissues were paralleled by those obtained in cultured *Fmr1*KO neurons. Although we found several commonalities, there were systematic differences that were surprising to us and, to our knowledge, are previously unreported. For example, in the in situ CNS of the *Fmr1*KO mouse, there was a significant enrichment of genes involved in immunological signaling; this pattern was not evident in cultured neurons. Conversely, in cultured neurons, we found a perturbation in several overlapping excitatory glutamatergic signaling cascades. Most are downstream of mGluR and neurotrophin signaling and may be coupled to FMRP-regulated mRNA translation.

As indicated by PCA, the distance between KO and WT clusters in cultures was higher than the distance between KO and WT in intact embryonic brain tissue. In addition, we observed a drastic difference in the numbers of differentially regulated genes in cultured cells and intact brain. Both findings suggest that the murine gene expression profile of cortical neuronal cultures and hippocampal neuronal cultures captures the difference between FXS and WT better than brain tissue. One possible explanation for this finding is that the effects of the *Fmr1* mutation on gene expression are more directly observable in cultured cells because of the more simplistic nature of the model with fewer different cell types and absence of a buffer to perturbation (culture). Primary brain is a complex tissue that can develop compensatory mechanisms to disease and has many cell types, with different gene expression patterns, therefore diluting a particular phenotype. The phenotype is exaggerated in culture and subtle in the organism, with factors not present in vitro playing a role in disease pathogenesis and severity.

The findings in our cultured cells were congruent with the well-established classical mGluR theory of FXS [1, 12, 13]. Specifically, we found significant perturbation in LTP, neurotrophin signaling pathway, and mammalian target of rapamycin (mTOR) and mitogen-activated protein kinase (MAPK) signaling cascades in *Fmr1*KO cultures. Over-activation of these cascades could lead to abnormal synaptic function owing to exaggerated protein synthesis in FXS [2, 4]. Of the differentially expressed genes, several are key activators in these aforementioned pathways [2]. Several have also been implicated in autism-related disorders [26], which have a high rate of comorbidity with FXS. However, as a large percentage (15–60 %) of children with FXS meet diagnostic criteria for autism, the findings might be relevant to a specific subset of cases with autism. Our findings support the hypothesis that the transcriptional variation in this Fragile X model is focused within mGluR-activated pathways related to synaptic plasticity.

Although the contrast between gene expression in brain tissue and in cultured neurons in *Fmr1*KO is novel, the finding of an immunological signal in the CNS is not. A module enriched for immune genes and glial markers has been observed in autism spectrum disorder (ASD) human post-mortem brain tissue [27, 28], and immunity response-related pathways were perturbed in *Fmr1*KO and *Tsc2*+/− murine cerebellum [29]. Various studies have implicated a dysregulated immune system response in ASD [30–35], but the exact mechanisms by which this dysfunction relates to autism-related disorders are not well-established. Moreover, although the genes are labeled as immunological in function, they also function as morphogens in the developing CNS [36]. Thus, their appearance in the intact CNS may mark a change in development rather than the inflammatory response they evince in the peripheral vasculature.

One possible explanation is that the end result of the *FMR1*-related disorders is an immunological phenotype that can be observed in the brain. However, culturing cells frees them from environmental factors that influence this phenotype, allowing them to manifest the synaptic-related dysfunctions of FXS. In addition, one of the reasons why synaptic and/or neuronal effects were more prominent in neuronal cultures may be because the immunological changes are less visible due to the lack of blood vessels or limited number and type of glia in cultured neurons. Another explanation is the presence of active glial cells in brain—especially microglia, which are the brain’s professional phagocytes. Transformation of microglia to reactive states in response to pathology has been known for decades as microglial activation [37]. Neuroglial activation and neuroinflammation have been observed in the brains of autism patients [28, 35, 38–40] and reactive astrocytes in several brain regions of *Fmr1*KO mice were previously revealed [41]. Moreover, the morphological phenotype observed in *Fmr1*KO neurons—the high density of dendritic spines [8–11]—might be linked to pruning defects by microglial cells [42–45]. The immunological phenotype therefore adds to prior evidence of a link between CNS pathology and glial activation [46, 47] in *Fmr1*KO mice [41].

We used Human Brain Transcriptome data to construct a map of genes that are active at different stages of human brain development. We defined three clusters of genes corresponding to prenatal, early, and late developmental time points in the cortex and hippocampus. We highlighted human genes homologous to the differentially expressed murine genes from culture and brain tissue, providing a temporal analysis of the expression of these genes. Up-regulated genes in KO cultures (as compared to WT cultures) tended to be genes whose physiological expression peaks early in healthy development. By contrast, differentially expressed genes in brain tissues were those whose expression peaks later in healthy development. Up-regulated genes from intact KO murine hippocampus and cortex were over-represented among genes with later physiological expression in human development. We hypothesize that the isolation and culture of neurons caused cortex and hippocampus cells to act “juvenile” or “de-differentiated” and therefore to express a “younger” transcriptomic profile. We have also found an enrichment of down-regulated genes in *Fmr1*KO cultures in literature databases on cognitive traits (MGI murine genes associated with behavioral/neurological phenotype, human SFARI genes, and FMRP binding targets). Together, we speculate that KO cultures bear a greater resemblance to earlier stages of development, and conversely, primary brain tissue bears a stronger resemblance to later stages of development.

## Conclusions

The findings from this study are summarized in Table 3 and highlighted in the list below:

**Table 3:** Summary of findings

	Differentially expressed genes in *Fmr1*KO vs WT (disease genes)			
	Culture		Brain	
	Hippocampus	Cortex	Hippocampus	Cortex
Number of differentially expressed genes	2648	3372	726	866
Distance between KO and WT clusters on PCA (PC1/PC2)	3.73/0.7	3.31/3.20	0.99/0.6	0.39/0.32
Pathway enrichment	Neuronal processes		Immune processes	
Human development	Early (H0 cluster)	Early (C0 cluster)	Later (H1 cluster)	Later (C2 cluster)
	Up-regulated	Up-regulated	Down- and up- regulated	Up-regulated
SFARI human genes	Significantly enriched in down-regulated genes	Significantly enriched in down-regulated genes	Not significant	Not significant
MGI mouse genes associated with behavioral/neurological phenotype	Significantly enriched in down-regulated genes	Significantly enriched in down-regulated genes	Not significant	Not significant
FMRP targets	Significantly enriched in down-regulated genes	Significantly enriched in down- and up- regulated genes	Not significant	Not significant

1. There is an over-representation of immune-related transcriptional activity in embryonic *Fmr1*KO cortex and hippocampus in comparison to neuronal signaling pathways in cortical and hippocampal primary cultures.
  Importance: In addition to neuronal pathology, research on disease treatments should also consider the impact of immune dysregulation, which is comparatively less studied.
2. Up-regulated genes in murine KO cultures corresponded to genes whose peak expression occurs *early* in human development, while differentially expressed genes measured in murine KO embryonic brain tissue corresponded to genes whose peak expression occurs *later* in human development.
  Importance: Cultured cells may recapitulate an early phase of the disease, which is also less obscured with “immunological” phenotype and in vivo compensatory mechanisms.
3. Down-regulated differentially expressed genes in KO cultures are enriched for (1) genes implicated in autism (SFARI), (2) genes associated with behavioral/neurological phenotypes of autism in mice (MGI), and (3) FMRP binding targets.
  Importance: These findings suggest that there are potential shared disease-related mechanisms in fragile X and other autism phenotypes in mice.
4. Together, these results suggest that the *Fmr1*KO culture system:
  Better captures the difference between genotypes in comparison to embryonic brain tissue.
  Bears greater resemblance to earlier stages of human development
  Bears the significant resemblance to the genes previously implicated with autism in human in comparison to brain tissue

To conclude, cultured hippocampal and cortical cells exhibited a more neurogenesis-related gene transcriptome, while primary brain tissue exhibited a more immunological transcriptome. These results are consistent with previous findings showing a role for the immunological response in neurodevelopmental disorders. *Fmr1*KO differentially expressed genes in culture coincided with genes active in early human sdevelopment, while the brain-related differentially expressed genes coincided with genes more active later in human development.

## Availability of supporting data

The data set supporting the results of this article is available in the Gene Expression Omnibus repository with the accession identifier GSE71034 (http://www.ncbi.nlm.nih.gov/geo/query/acc.cgi?acc=GSE71034).

## Additional files

Additional file 1: Table S1. Genes associated with defects in cognitive development: MGI, SFARI, and FMRP targets. (XLSX 111 kb) Additional file 2: Table S2. Centroid calculation for each cluster in Fig. 1. The distance between clusters is represented as a difference of absolute values on the PC1 and PC2 axes. (XLSX 9 kb) Additional file 3: Table S3. List of significant differential gene expressed in four systems. (XLSX 1 mb) Additional file 4: Table S4. Detailed KEGG pathway-enrichment analysis of differentially expressed genes between KO and WT mice. (XLSX 21 kb) Additional file 5: Table S5. Selected over-represented KEGG pathways with core differentially expressed genes in *Fmr1*KO that made a pathway significant. (XLSX 11 kb) Additional file 6: Figure S1. Metacore enrichment analysis of differentially expressed genes in culture and in primary brain based on differentially affected functional ontologies such as (A) “Process Networks” and (B) “Diseases (by Biomarkers)”. (DOCX 124 kb) Additional file 7: Table S7. Overlap of pathways and commonalities between two different types of preparations (brain and culture) and between two different brain regions (cortex and hippocampus). (XLSX 237 kb) Additional file 8: Table S8. Unique and annotated mouse genes after differential gene expression analysis in four systems. Mapping to MGI genes, human SFARI genes, and FMRP targets. (XLSX 4 mb) Additional file 9: Table S9. Significance of overlap for hippocampal or cortical cultures and embryonic brain tissues of KO/WT up/down/combine differentially expressed genes AND MGI genes with behavioral/neurological phenotype (http://www.informatics.jax.org/searches/Phat.cgi?id=MP:0005386). Fisher’s exact test was used to calculate the statistics. (XLSX 11 kb) Additional file 10: Table S10. Significance of overlap for hippocampal or cortical cultures and embryonic brain tissues of KO/WT up/down/combine differentially expressed genes AND human SFARI genes. Fisher’s exact test was used to calculate the statistics. (XLSX 12 kb) Additional file 11: Table S11. Significance of overlap for hippocampal or cortical cultures and embryonic brain tissues of KO/WT up/down/combine differentially expressed genes AND FMRP binding targets. Fisher’s exact test was used to calculate the statistics. (XLSX 12 kb) Additional file 12: Table S12. Integration of mouse and human data to represent development. Final number of genes in common between the datasets: 13830. (XLSX 2 mb) Additional file 13: Table S13. Significance of overlap for murine differentially expressed genes (up + down) and genes represented in a specific cluster in human development. Fisher’s exact test was used to calculate the statistics. (XLSX 16 kb) Additional file 14: Table S14. Enriched pathways in murine differentially expressed up-regulated genes in cortical and hippocampal cultures highlighted on human development (C0 and H0 clusters respectively). (XLSX 11 kb)

## Abbreviations

ASD: autism spectrum disorder
CAMs: cell adhesion molecules
CNS: central nervous system
DAVID: Database for Annotation, Visualization and Integrated Discovery
*Fmr*1: fragile X mental retardation 1
FMRP: fragile X mental retardation protein
FXS: fragile X syndrome
HBT: human brain transcriptome
HIP: hippocampus
KEGG: Kyoto Encyclopedia of Genes and Genomes
KO: knockout
LTD: long-term depression
LTP: long-term potentiation
MAPK: mitogen-activated protein kinase
MGI: Mouse Gene Informatics
mGluR: metabotropic glutamate receptor
mTOR: mammalian target of rapamycin
NCX: neocortex
PC: principal component
PCA: principal component analysis
RMA: robust multi-array average
SFARI: Simons Foundation Autism Research Initiative
Tsc2: tuberous sclerosis protein 2
WT: wild type
