# Supplementary material for: Gene expression analysis in Fmr1KO mice identifies an immunological signature in brain tissue and mGluR5-related signaling in primary neuronal cultures
Source: Mol Autism. 2015 Dec 21;6:66. doi: 10.1186/s13229-015-0061-9 (PMC4687343; doi:10.1186/s13229-015-0061-9)
Supplement: Additional file 6: Figure S1. — Metacore enrichment analysis of differentially expressed genes in culture and in primary brain based on differentially affected functional ontologies such as (A) “Process Networks” and (B) “Diseases (by Biomarkers)”. (DOCX 124 kb) [file 13229_2015_61_MOESM6_ESM.docx]

Figure S1: Metacore enrichment analysis of differentially expressed genes in culture and in primary brain based on differentially affected functional ontologies such as (A) “Process Networks” and (B) “Diseases (by Biomarkers)”.

| 1. |  | HippoCultureKOminusWT_EntrezID_genelist |
| --- | --- | --- |
| 2. |  | CorticalCultureKOminusWT_EntrezID_genelist |
| 3. |  | HippoBrainKOminusWT_EntrezID_genelist |
| 4. |  | CortexBrainKOminusWT_EntrezID_genelist |


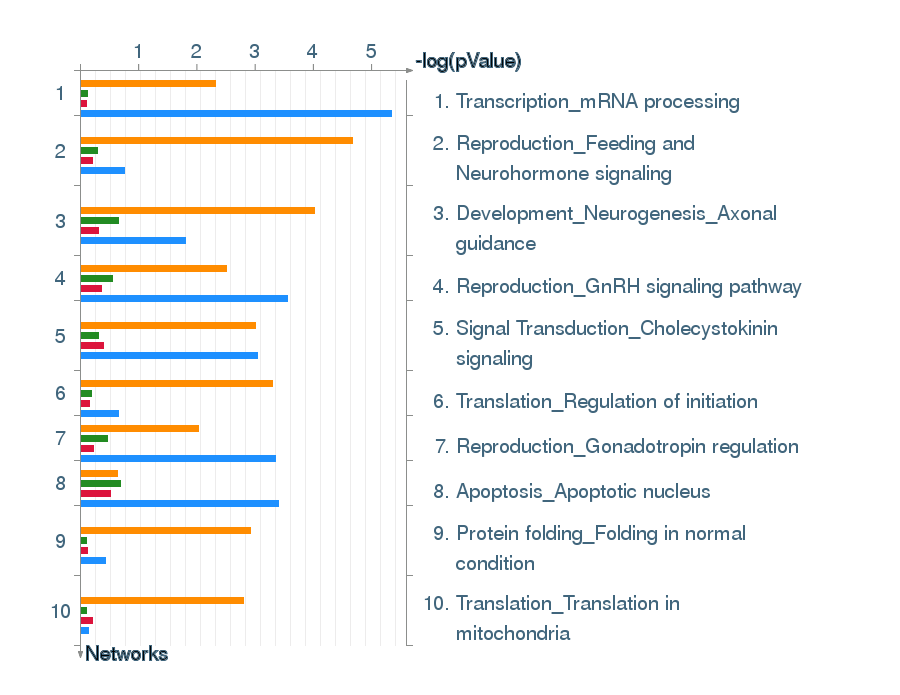


**A.**


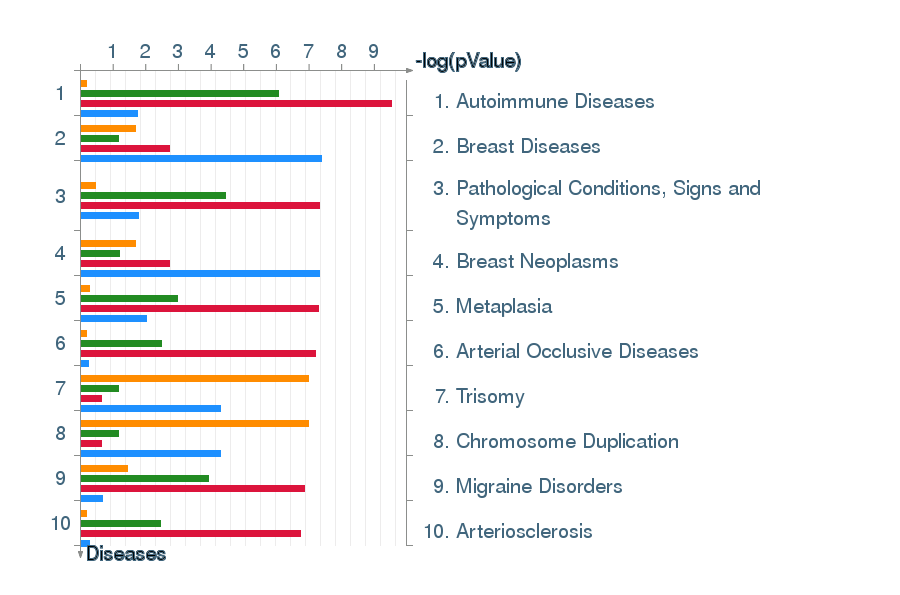


**B.**
